# Cirrhosis, a Global and Challenging Disease

**DOI:** 10.3390/jcm11216512

**Published:** 2022-11-02

**Authors:** José Ignacio Fortea, Javier Crespo, Ángela Puente

**Affiliations:** Gastroenterology and Hepatology Department, Clinical and Translational Research in Digestive Diseases, Valdecilla Research Institute (IDIVAL), Marqués de Valdecilla University Hospital, 39011 Santander, Spain

Cirrhosis is the result of sustained liver damage leading to the diffusion of hepatic fibrosis, wherein the normal hepatic architecture is replaced by abnormally organized nodules separated by fibrous septa that connect the different vascular structures of the hepatic lobule [1]. Chronic viral hepatitis (i.e., hepatitis B or C infection), alcohol-related liver disease, and non-alcoholic fatty liver disease (NAFLD) are the most common causes [2]. Whatever the etiology, the majority of its complications are due to the development of portal hypertension [3]. Cirrhosis is a heterogeneous disease with two main prognostic stages: the compensated stage with a median survival exceeding 12 years, defined by the absence of present or past complications of cirrhosis, and the decompensated stage, marked by the presence of overt ascites or hepatic hydrothorax, variceal bleeding, and overt hepatic encephalopathy, with median survival dropping to 2–4 years [4].

The global impact of cirrhosis on the public health is high, although not evenly distributed across the world with the poorest countries bearing the greatest burden [5]. In the most recent Global Burden of Disease study, from 2019, cirrhosis is the 10th most common cause of death, the fifth leading cause of death in people aged 50–69 years, and together with liver cancer, accounts for 3.5% of all deaths worldwide [6] (data can be accessed through this website: http://www.healthdata.org/gbd/2019, accessed on 29 October 2022). In Europe, liver disease is now the second leading cause of years of working life lost after only ischemic heart disease [2]. In regard to primary liver cancer, which usually develops in patients with cirrhosis, a recent study estimated that 830,200 people died from liver cancer globally in 2020, being among the top three causes of cancer death in 46 countries. Moreover, it was the second most common cause of premature death from cancer in 2020, after lung cancer, and the authors estimated that the number of new cases and deaths from liver cancer could rise by >55% by 2040 [7]. Despite this burden on public health and finances, cirrhosis has not been considered a major health issue. For example, the World Health Organization (WHO) assigns zero disability to compensated cirrhosis and considers decompensated cirrhosis as only mildly disabling [5]. Moreover, the term ‘liver disease’ is not included in the WHO list of non-communicable diseases. This lack of appropriate consideration coupled with the perception that the disease is largely related to alcohol consumption contributes to a low public awareness of the relevance of cirrhosis and leads to late diagnosis and missed opportunities to mitigate causative factors and prevent subsequent progression [1]. Fortunately, initiatives such as the EASL–Lancet Liver Commission are being made to counteract the stigmatization of the disease and promote early disease detection and liver health [2].

The special Issue titled “The Clinical Management of Liver Cirrhosis: Current Concepts, Recent Advances and Future Trends” includes seven manuscripts (four original papers, a meta-analysis and two reviews) that focus on several important topics and complications of cirrhosis. Two of them address the issue of non-invasive tests (NITs) for evaluation of liver fibrosis. The universal implementation of NITs in clinical practice has significantly reduced the number of liver biopsies, which are now mainly indicated to determine the cause of liver disease in selected cases, and not to stage fibrosis [1]. NITs can be classified into blood-based tests (e.g., Fibrosis Score 4), methods assessing physical properties of the liver tissue (e.g., liver stiffness) and imaging methods assessing the anatomy of the liver and other abdominal organs [8]. Fujita et al., focus their review on serum biomarkers [9]. Advantages of this type of NIT are their wide availability and applicability, good reproducibility, and no cost (except for patented markers) [8]. These tests are increasingly used in primary care to stratify the risk of advanced fibrosis in high-risk individuals, allowing early referral of patients to a specialist liver center [2]. Of note, the etiology of the disease and confounding factors (e.g., liver and systemic inflammation) must be taken into account when interpreting their results [8]. In the other study, Yano et al. compared the accuracy of hepatic extracellular volume fraction (fECV) analysis with contrast-enhanced computed tomography (CT) with magnetic resonance (MR) elastography in staging liver fibrosis [10]. The fECV expands with fibrosis deposition and this technique uses the contrast agents of CT and MR to evaluate this fECV expansion, which has been shown to correlate with fibrosis stage in a few studies [11]. In their single-center retrospective study, the authors did confirm this correlation, but the diagnostic accuracy of fECV analysis using CT was lower than MR elastography and biased by liver inflammation [10].

The remaining three original manuscripts focus on two well-known complications of cirrhosis such as acute kidney injury (AKI) and hepatic hydrothorax, and the other on osteoporosis, a complication often overlooked in this population. AKI is highly prevalent among hospitalized patients with cirrhosis (up to 47%) and is associated with a 7-fold increase in mortality [12]. Once AKI is diagnosed, it is essential to differentiate between acute tubular necrosis AKI (ATN-AKI) and hepatorenal syndrome (HRS)-AKI. Biomarkers of renal tubular injury can help in the differential diagnosis, although they are not clinically available in most of the centers [13]. They include tubular proteins released during cell damage (e.g., N-acetyl-β-D-glucosaminidase (NAG)), tubular proteins upregulated by injury (e.g., kidney injury molecule 1 and neutrophil gelatinase–associated lipocalin (NGAL)) and markers of inflammation such as interleukin-18. In general, the higher these biomarkers are, the more likely the patient is to have ATN, being NGAL the most studied biomarker [12,13]. In their multicenter, prospective study including 262 patients with AKI (73 HRS-AKI), Yoo et al. aimed to evaluate the usefulness of urine NAG for predicting the response to terlipressin in patients with HRS-AKI and the transplant-free survival in the whole cohort. Despite providing prognostic information, urine NAG was not able to predict the response to terlipressin [14]. Of note, levels of NAG were greater in patients with HRS-AKI than in patients with ATN-AKI, a finding that raises concern regarding the accurate diagnosis of each type of AKI.

Hepatic hydrothorax (HH) is another complication of cirrhosis associated with high mortality. It is defined as the presence of pleural effusion in a patient with cirrhosis without evidence of underlying cardiopulmonary disease and occurs in up to 5–12% of the patients [15,16,17]. Most cases occur in patients with ascites and are unilateral and right-sided, since the diaphragmatic defects are more frequent on the right side of the diaphragm, which is thinner and less muscular, and thus, more prone to collagen fiber deterioration [15]. In their single-center retrospective study, including a total of 763 patients with cirrhosis and ascites admitted in a tertiary care facility, Matei et al. confirm the negative prognostic impact of HH in decompensated cirrhotic patients using propensity score matching to adjust for confounding variables [18]. These results, along with previous literature, should alert clinicians to consider liver transplantation earlier in these patients [17,19].

A more prevalent but often ignored complication of cirrhosis is osteoporosis, which occurs in about 30% of patients with a higher prevalence in the presence of related risk factors (e.g., cholestatic liver diseases, advanced liver disease, postmenopausal women, or corticosteroid therapy) [20]. Moreover, improvements in the management of bone health in transplanted patients have reduced the incidence of fractures. Hence, current guidelines recommend screening for osteoporosis and osteopenia in patients with cirrhosis with bone mineral density (BMD) measured by lumbar and femoral densitometry [20]. The cross-sectional study by Ogiso et al., including 275 patients with cirrhosis aimed to determine whether trabecular bone score (TBS) can identify patients at risk of vertebral fractures. TBS is highly correlated, albeit indirectly, with three-dimensional parameters of bone microarchitecture and has been shown to predict osteoporotic fractures in primary osteoporosis and some secondary osteoporosis, independent of BMD [21]. The authors found that TBS was independently associated with the presence of vertebral fractures and correlated with BMD. These findings need to be validated in larger prospective studies to determine whether we should incorporate TBS as a complementary tool to BMD to assess the risk of osteoporotic fractures in patients with cirrhosis.

The meta-analysis by Castellana et al., aimed to determine the prevalence of the absence of cirrhosis in subjects with non-alcoholic fatty liver disease (NAFLD)-associated hepatocellular carcinoma (HCC) [22]. This is an important issue, since current guidelines either do not give a specific recommendation or do not recommend screening of HCC in patients with non-cirrhotic NAFLD [23,24,25,26]. The authors included 30 original articles reporting data on the presence or absence of cirrhosis among at least 50 subjects with NAFLD-associated HCC. Among the 13,371 subjects evaluated, the pooled prevalence of non-cirrhotic NAFLD-associated HCC was 37%, with heterogeneity among the studies. The prevalence appeared to be higher in Asia versus Europe, North America and South America, as well as in studies adopting histology only as the reference standard for the diagnosis of cirrhosis compared to other modalities. These results are relevant, and the authors should be congratulated for their effort. However, this meta-analysis does not overcome the limitations of previous related meta-analyses [27,28]. Indeed, the studies included were mostly retrospective and differ in terms of methodology, population characteristics, and tools used for HCC and NAFLD diagnosis. Moreover, since HCC screening is not recommended in these patients, the available data are based on incidental cases. For all these reasons, we agree with the authors that routine surveillance for HCC in all patients with non-cirrhotic NAFLD cannot be recommended on the basis of their results and that further studies on this topic are urgently needed.

The last paper of this Special Issue is a review made by our group on portal vein thrombosis (PVT) in patients with cirrhosis [29]. PVT is the most common thrombotic event in this population, with increased rates in the setting of advanced liver disease. Despite being a well-known complication, the contribution of PVT to hepatic decompensation and overall mortality is still a matter of debate. The lack of large prospective observational studies and randomized trials explain the heterogenous diagnostic and therapeutic recommendations of current guidelines [4,30].

In conclusion, this Special Issue encompasses several important topics and complications of cirrhosis. Both of the reviews make an updated synopsis of serum NITs and PVT in the setting of cirrhosis, while the original studies provide relevant new data on each of their respective topics, further fueling the need for additional studies to bridge the gaps in knowledge.
